# A Pseudo-Random Beamforming Technique for Improving Physical-Layer Security of MIMO Cellular Networks

**DOI:** 10.3390/e21111038

**Published:** 2019-10-25

**Authors:** Woong Son, Han Seung Jang, Bang Chul Jung

**Affiliations:** 1 Department of Electronics Engineering, Chungnam National University, Daejeon 34134, Korea; woongson@cnu.ac.kr; 2 School of Electrical, Electronic Communication and Computer Engineering, Chonnam National University, Yeosu 59626, Korea

**Keywords:** pseudo-random beamforming, beam selection, physical-layer security, secrecy capacity, user scheduling, opportunistic feedback

## Abstract

In this paper, we propose a pseudo-random beamforming (PRBF) technique for improving physical-layer security (PLS) in multiple input multiple output (MIMO) downlink cellular networks consisting of a legitimate base station (BS), multiple legitimate mobile stations (MSs) and potential eavesdroppers. The legitimate BS can obtain available potential eavesdroppers’ channel state information (CSI), which is registered in an adjacent cell. In the proposed PRBF technique, the legitimate BS *pseudo-randomly* generates multiple candidates of the transmit beamforming (BF) matrix, in which each transmit BF matrix consists of multiple orthonormal BF vectors and shares BF information with legitimate MSs before data transmission. Each legitimate MS generates receive BF vectors to maximize the receive signal-to-interference-plus-noise (SINR) for all pseudo-randomly generated transmit beams and calculates the corresponding SINR. Then, each legitimate MS sends a single beam index and the corresponding SINR value of the BF vector that maximizes the received SINR for each BF matrix since a single spatial stream is sent to each legitimate MS. Based on the feedback information from legitimate MSs and the CSI from the legitimate BS to eavesdroppers, the legitimate BS selects the optimal transmit BF matrix and the legitimate MSs that maximizes secrecy sum-rate. We also propose a codebook-based opportunistic feedback (CO-FB) strategy to reduce feedback overhead at legitimate MSs. Based on extensive computer simulations, the proposed PRBF with the proposed CO-FB significantly outperforms the conventional random beamforming (RBF) with the conventional opportunistic feedback (O-FB) strategies in terms of secrecy sum-rate and required feedback bits.

## 1. Introduction

Security of wireless communication has received much attention from both academia and industry. Secure transmission is significantly important especially for military communications. To define the degree of security of communications, the concept of physical-layer security (PLS) was first defined in [1], and *secrecy capacity* has been used as a metric for PLS performance evaluations, which is defined as the difference between the channel capacity of authorized and unauthorized communication links [2,3,4,5].

Recent studies for improving PLS were reviewed and summarized in various multi-user wireless networks environments such as single-user single-antenna wire-tap channel, single-user multi-antenna wire-tap channel, wire-tap broadcast channel, wire-tap multiple-access channel, wire-tap interference channel, wire-tap relay and cooperative channels, etc. [6]. Several user scheduling algorithms combined with a beamforming (BF) technique were proposed for multi-user wireless networks for enhancing secrecy capacity. Jin et al. [7] showed that the optimal multi-user diversity can be obtained with a threshold-based user scheduling algorithm in a single-cell single-input single output (SISO) uplink wiretap network. In his another study [8], the threshold-based user scheduling algorithm for multi-cell SISO uplink wiretap networks was proposed. An artificial noise (AN)-aided opportunistic user scheduling algorithm was recently proposed for a single multi-user SISO uplink wiretap network with multiple eavesdroppers, where non-scheduled users generate AN in order to improve the PLS [9]. Even though there exist many recent BF techniques with an opportunistic user scheduling algorithm for a single-cell downlink wire-tap network in the literature, most recent studies assumed a single antenna at legitimate MSs and eavesdroppers.

Below are some of recent studies on multi-antenna based BF techniques for improving PLS. In [10], when an imperfect channel state information (CSI) between the legitimate base station (BS) and the eavesdropper is assumed, on–off opportunistic BF technique based on *statistical* CSI was proposed in a single-cell multi-user multiple-input single-output (MISO) downlink wire-tap network. In addition, a random beamforming (RBF) technique was proposed to maximize secrecy sum-rate in a single-cell multi-user MISO downlink network, where the legitimate BS selects a subset of active beams according to system parameters such as the number of users in a cell, the number of transmit antennas at the legitimate BS, and wireless channel conditions [11]. A RBF technique with variable number of active beams, which is similar to the one in [11], was also proposed to minimize secrecy *outage* capacity in a single-cell multi-user MISO downlink network [12]. A maximum signal-to-leakage-and-noise ratio (SLNR)-based BF technique was proposed in multiple input multiple output (MIMO) downlink wiretap networks [13], and, based on SLNR and zero-forcing technique, the BF matrix can be designed to increase the secrecy capacity.

Eavesdroppers are generally defined as passive or active eavesdroppers with respect to their eavesdropping strategies. A passive eavesdropper attempts to eavesdrop the data transmission without another operation [14,15,16]. However, an active eavesdropper attempts to eavesdrop the data transmission using fake information feedback [15,16]. Some eavesdroppers can also generate jamming signals, interfering with the data transmission of legitimate links [17,18,19,20,21]. They are called potential eavesdroppers, are registered in another cell but unauthorized in the legitimate cell, and can be classified as active eavesdroppers [22,23,24,25]. There are some related studies with respect to potential eavesdroppers. In [22], an orthogonal RBF technique with a opportunistic user scheduling algorithm was proposed to improve PLS in a single-cell MISO downlink cellular network where it is assumed that each legitimate MS is wire-tapped by an eavesdropper as a worst-case secrecy scenario. In particular, the authors called the eavesdroppers registered (but unintended) on the legitimate network. In addition, the eavesdroppers are obligated to feed their signal-to-interference-plus-noise ratio (SINR) values to the legitimate BS. Therefore, the authors considered a system model in which potential eavesdroppers exist. In [23], the authors considered potential eavesdropper that have a shorter access distance than legitimate receivers due to wireless channel attenuation. In secrecy wireless information and power transfer (SWIPT) systems, the legitimate transmitter can exploit near potential eavesdroppers’ CSI since they are legitimate devices for harvesting power. Then, based on potential eavesdroppers’ CSI, the legitimate transmitter can properly transmit to maximize PLS performance requirement of energy harvesting. In [24], the public access point (AP) for downlink transmission does not know which users are eavesdroppers. However, the AP considers only one legitimate user, which is selected for downlink transmission, and the other unscheduled users as potential eavesdroppers. In addition, the authors assumed the non-colluding eavesdroppers model (i.e., the non-cooperative potential eavesdroppers assumption). They also assumed that the secrecy rate only depends on the best CSI among potential eavesdropper. All users’ CSIs are estimated at AP by received packet from users. Then, the AP generates the BF vector based on all estimated CSIs for secure transmission. In [25], the authors considered a multi-user SISO uplink wire-tap network consisting of multiple users with a single antenna and multiple potential eavesdropper with a single antenna. Similar to [24], the authors considered a non-colluding eavesdroppers model. Thus, they considered only one potential eavesdropper, which has best CSI from the scheduled legitimate user. They proposed the optimal user scheduling and threshold-based user scheduling for PLS enhancement and analyzed the secrecy rate according to the proposed scheduling scheme.

There are some related studies with respect to beamforming algorithms for PLS enhancement.

- In [13], the authors considered two wire-tap channel models in different users condition. In multiple-input single-output multi-eavesdropping antennas (MISOME) wiretap network, the authors assumed that all of the wireless channel matrices are known to the legitimate sender with multiple antennas and legitimate receiver with a single antenna. Otherwise, in multi-user multiple-input single-output multi-eavesdropping antennas (MU-MISOME) wire-tap network, the authors also assumed that all of wireless channel matrices are known to the legitimate sender with multiple antennas and multiple legitimate receivers with a single antenna. However, an eavesdropper with multiple antennas only knows the wireless channel matrices from legitimate sender in the above system models. The authors proposed some beamforming algorithms for improving PLS such as a maximum-SLNR-based beamforming algorithm and a zero-forcing beamforming algorithm based on the eavesdropper’s CSI in MISOME and MU-MISOME wire-tap networks.
- In [26], the authors originally proposed a novel pseudo-random beamforming (PRBF) technique to maximize the achievable sum-rate in multi-cell downlink cellular networks. Each cell has a BS with multiple antennas and multiple MSs with a single antenna. By announcing an optimal BF candidate among multiple candidates of pseudo-randomly generated BF matrices at the BS coordinator, the multi-cell downlink sum-rate is maximized.
- In [27], the authors proposed a PRBF technique to improve PLS in single-cell downlink cellular networks. In addition, the authors assumed a system model consisting a legitimate BS with multiple antennas, multiple legitimate MSs with a single antenna and a potential eavesdropper with a single antenna. To maximize the achievable secrecy sum-rate in downlink cellular networks consisting of legitimate MSs with a single antenna and a potential eavesdropper, the PRBF technique based on legitimate MSs’ feedback information and a potential eavesdropper’s CSI is proposed.

The main contributions of this paper are summarized as follows:

- We investigate the secrecy sum-rate in single-cell MIMO downlink cellular networks consisting of a legitimate BS with multiple antennas, legitimate MSs with multiple antennas and eavesdroppers with multiple antennas.
- We also consider the conventional F-FB, opportunistic feedback (O-FB) and newly proposed the codebook-based opportunistic feedback (CO-FB) strategy.
- In addition, we compare the conventional F-FB, O-FB [26,27] and the proposed CO-FB in terms of secrecy sum-rate and required feedback bits (feedback overhead).

The remainder of this paper is organized as follows. In Section 2, we describe the system model of MIMO downlink celluar networks with eavesdroppers In Section 3, we explain the overall procedure of the proposed PRBF technique and also compare the conventional F-FB and O-FB with the proposed CO-FB. Computer simulation results are presented in Section 4. Finally, the conclusions are briefly drawn in Section 5.

## 2. System Model

**Figure 1:**
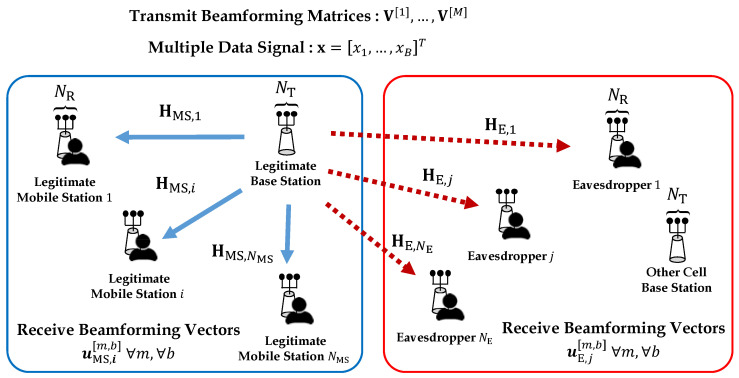
System model of MIMO downlink cellular network.

Let us consider a TDD MIMO downlink network consisting of a legitimate BS with $N_{T}$ antennas, $N_{\mathrm{MS}}$ legitimate MSs with $N_{R}$ antennas, and $N_{E}$ eavesdroppers with $N_{R}$ antennas, as shown in Figure 1.

We assume that the legitimate BS is in the blue box and other cell BSs are in the red box. In particular, legitimate MSs existing in other cells (marked with a red box) can become *potential eavesdroppers* who are unauthorized MSs for the legitimate network in the blue box. We assume the CSI of potential eavesdroppers is available at the legitimate BS, which implies that the legitimate BS is assumed to know the wireless channel from itself to the potential eavesdroppers. This is possible by *overhearing* the pilot signals from the potential eavesdroppers when the eavesdroppers send packets to their own BSs in TDD systems. Thus, each BS can estimate all the channel coefficients from not only the MSs belonging to itself but also the MSs in other cells, which is also known as *local CSI* assumption. Many studies on multi-cell MIMO networks assume the local CSI as well [28,29,30]. In addition, many previous studies on physical-layer security assume that the legitimate communication nodes know the wireless channel to the eavesdroppers based on local CSI assumption [7,8,25,27]. In addition, we assume that the same frequency band is used for data transmission. All devices are affected by the interference caused by the desired signal from other cells. *M* candidates of transmit BF matrix are *pseudo-randomly* generated at the legitimate BS. Then, *M* candidates of transmit BF matrix are represent as $V^{\left[1\right]},\ldots ,V^{\left[M\right]}$. The *m*th transmit BF matrix is denoted by $V^{\left[m\right]}=\left[v^{\left[m,1\right]},\ldots ,v^{\left[m,b\right]},\ldots ,v^{\left[m,B\right]}\right]\in C^{N_{T}\times B}$, where m∈M≜{1,…,M} and $b\in B≜\{1,\ldots ,B\left(=N_{T}\right)\}$. $v^{\left[m,b\right]}\in C^{N_{T}\times 1}$ represents the *b*th transmit BF vector in the *m*th transmit BF matrix. Corresponding to MB transmit BF vectors, each legitimate MS generates MB receive BF vectors based on MMSE. Then, *M* candidates of receive BF matrix at the *i*th legitimate MS are represented as $U_{\mathrm{MS},i}^{\left[1\right]},\ldots ,U_{\mathrm{MS},i}^{\left[M\right]}$, where $i\in N_{\mathrm{MS}}≜\left\{1,\ldots ,N_{\mathrm{MS}}\right\}$. The *m*th receive BF matrix at the *i*th legitimate MS is represented as $U_{\mathrm{MS},i}^{\left[m\right]}=\left[u_{\mathrm{MS},i}^{\left[m,1\right]},\ldots ,u_{\mathrm{MS},i}^{\left[m,b\right]},\ldots ,u_{\mathrm{MS},i}^{\left[m,B\right]}\right]\in C^{N_{R}\times B}$. In addition, $u_{\mathrm{MS},i}^{\left[m,b\right]}\in C^{N_{R}\times 1}$ represents the *b*th receive BF vector in the *m*th receive BF matrix at the *i*th legitimate MS. Similarly, each eavesdropper also generates MB receive BF vectors corresponding MB transmit BF vectors based on MMSE. This assumption is reasonable to consider the worst-case in terms of PLS of legitimate devices. Then, *M* candidates receive BF matrix at the *j*th eavesdropper are represented by $U_{E,j}^{\left[1\right]},\ldots ,U_{E,j}^{\left[M\right]}$, where $j\in N_{E}≜\left\{1,\ldots ,N_{E}\right\}$. The *m*th receive BF matrix at the *j*th eavesdropper is denoted as $U_{E,j}^{\left[m\right]}=\left[u_{E,j}^{\left[m,1\right]},\ldots ,u_{E,j}^{\left[m,b\right]},\ldots ,u_{E,j}^{\left[m,B\right]}\right]\in C^{N_{R}\times B}$. $u_{E,j}^{\left[m,b\right]}\in C^{N_{R}\times 1}$ denotes the *b*th receive BF vector in the *m*th receive BF matrix at the *j*th eavesdropper. $H_{\mathrm{MS},i}\in C^{N_{R}\times N_{T}}$ and $H_{E,j}\in C^{N_{R}\times N_{T}}$ denote the wireless channel matrix from the legitimate BS to *i*th legitimate MS and the wireless channel matrix from the legitimate BS to *j*th eavesdropper, respectively.

We assume that wireless channel components are independent and identically distributed (i.i.d.). In addition, we assume that wireless channel components are constant during one block (e.g., one frame), and large-scale fading components are equal to 1 from the legitimate BS to legitimate MSs and eavesdroppers. The legitimate BS transmits a data signal vector $x≜{\left[x_{1},\ldots ,x_{B}\right]}^{T}\in C^{B\times 1}$, which is satisfied by the power constraint $E\left[{\left|x\right|}^{2}\right]=P$. Without any loss of generality, the received signal vector $y_{\mathrm{MS},i}^{\left[m,b\right]}\in C^{N_{R}\times 1}$ at the *i*th legitimate MS with the *b*th transmit BF vector when the legitimate BS transmits a data signal vector x with the *m*th transmit BF matrix is given by

(1) $$\begin{array}{cc} y_{\mathrm{MS},i}^{\left[m,b\right]}= & H_{\mathrm{MS},i}V^{\left[m\right]}x+n_{\mathrm{MS},i}=H_{\mathrm{MS},i}v^{\left[m,b\right]}x_{b}+\sum_{l\neq b}^{B}H_{\mathrm{MS},i}v^{\left[m,l\right]}x_{l}+n_{\mathrm{MS},i}, \end{array}$$

where the additive thermal Gaussian noise vector at the *i*th legitimate MS is denoted by $n_{\mathrm{MS},i}\in C^{N_{R}\times 1}$ according to $\mathrm{CN}\left(0,N_{0}I_{N_{R}}\right)$.

The post-processed received signal ${\tilde{y}}_{\mathrm{MS},i}^{\left[m,b\right]}\in C$ with the *b*th receive BF vector in the *m*th receive BF matrix is given as

(2) $$\begin{array}{cc} {\tilde{y}}_{\mathrm{MS},i}^{\left[m,b\right]}= & {\left(u_{\mathrm{MS},i}^{\left[m,b\right]}\right)}^{H}y_{\mathrm{MS},i}^{\left[m,b\right]}\\ = & {\left(u_{\mathrm{MS},i}^{\left[m,b\right]}\right)}^{H}H_{\mathrm{MS},i}v^{\left[m,b\right]}x_{b}+{\left(u_{\mathrm{MS},i}^{\left[m,b\right]}\right)}^{H}\sum_{l\neq b}^{B}H_{\mathrm{MS},i}v^{\left[m,l\right]}x_{l}+{\left(u_{\mathrm{MS},i}^{\left[m,b\right]}\right)}^{H}n_{\mathrm{MS},i}\\ = & {\tilde{h}}_{\mathrm{MS},i}^{\left[m,b\right]}x_{b}+{\left(u_{\mathrm{MS},i}^{\left[m,b\right]}\right)}^{H}\sum_{l\neq b}^{B}h_{\mathrm{MS},i}^{\left[m,l\right]}x_{l}+{\tilde{n}}_{\mathrm{MS},i}, \end{array}$$

where the desired and interference signals at the *i*th legitimate MS are represented as the first and second terms on the right side of Equation (2), respectively. The post-processed additive thermal Gaussian noise at the *i*th legitimate MS follows ${\tilde{n}}_{\mathrm{MS},i}≜{\left(u_{\mathrm{MS},i}^{\left[m,b\right]}\right)}^{H}n_{\mathrm{MS},i}∼\mathrm{CN}\left(0,1\right)$. In this case, the post-processed effective channel ${\tilde{h}}_{\mathrm{MS},i}^{\left[m,b\right]}\in C$ at the *i*th legitimate MS is defined as

(3) $$\begin{array}{c} {\tilde{h}}_{\mathrm{MS},i}^{\left[m,b\right]}≜{\left(u_{\mathrm{MS},i}^{\left[m,b\right]}\right)}^{H}h_{\mathrm{MS},i}^{\left[m,b\right]}, \end{array}$$

where the effective channel vector is given by $h_{\mathrm{MS},i}^{\left[m,b\right]}≜H_{\mathrm{MS},i}v^{\left[m,b\right]}\in C^{N_{R}\times 1}$.

Similarly, the received signal vector $y_{E,j}^{\left[m,b\right]}\in C^{N_{R}\times 1}$ at the *j*th eavesdropper with the *b*th transmit BF vector when the legitimate BS transmits a data signal vector x with the *m*th transmit BF matrix is given by

(4) $$\begin{array}{cc} y_{E,j}^{\left[m,b\right]}= & H_{E,j}V^{\left[m\right]}x+n_{E,j}=H_{E,j}v^{\left[m,b\right]}x_{b}+\sum_{l=1,l\neq b}^{B}H_{E,j}v^{\left[m,l\right]}x_{l}+n_{E,j}, \end{array}$$

where the additive thermal Gaussian noise vector at the *j*th eavesdropper is denoted by $n_{E,j}\in C^{N_{R}\times 1}$ following $\mathrm{CN}\left(0,N_{0}I_{N_{R}}\right)$. The post-processed received signal ${\tilde{y}}_{E,j}^{\left[m,b\right]}\in C$ with the *b*th receive BF vector in the *m*th receive BF matrix is given as

(5) $$\begin{array}{cc} {\tilde{y}}_{E,j}^{\left[m,b\right]}= & {\left(u_{E,j}^{\left[m,b\right]}\right)}^{H}y_{E,j}^{\left[m,b\right]}\\ = & {\left(u_{E,j}^{\left[m,b\right]}\right)}^{H}H_{E,j}v^{\left[m,b\right]}x_{b}+{\left(u_{E,j}^{\left[m,b\right]}\right)}^{H}\sum_{l\neq b}^{B}H_{E,j}v^{\left[m,l\right]}x_{l}+{\left(u_{E,j}^{\left[m,b\right]}\right)}^{H}n_{E,j}\\ = & {\tilde{h}}_{E,j}^{\left[m,b\right]}x_{b}+{\left(u_{E,j}^{\left[m,b\right]}\right)}^{H}\sum_{l\neq b}^{B}h_{E,j}^{\left[m,l\right]}x_{l}+{\tilde{n}}_{E,j}, \end{array}$$

where the desired and interference signals at the *j*th eavesdropper are represented as the first and second terms on the right side of Equation (5), respectively. The post-processed additive thermal Gaussian noise at the *j*th eavesdropper follows ${\tilde{n}}_{E,j}≜{\left(u_{E,j}^{\left[m,b\right]}\right)}^{H}n_{E,j}∼\mathrm{CN}\left(0,1\right)$. In this case, the post-processed effective channel ${\tilde{h}}_{E,j}^{\left[m,b\right]}\in C$ at the *j*th eavesdropper is defined as

(6) $$\begin{array}{c} {\tilde{h}}_{E,j}^{\left[m,b\right]}≜{\left(u_{E,j}^{\left[m,b\right]}\right)}^{H}h_{E,j}^{\left[m,b\right]}, \end{array}$$

where the effective channel vector is given by $h_{E,j}^{\left[m,b\right]}≜H_{E,j}v^{\left[m,b\right]}\in C^{N_{R}\times 1}$.

## 3. Pseudo-Random Beamforming for Improving Physical-Layer Security

In this section, we explain the proposed technique for PLS enhancement in downlink cellular networks in detail. Pseudo-random beamforming algorithm for PLS enhancement, as shown in Algorithm 1. 

**Algorithm 1:** Pseudo-random beamforming algorithm for PLS enhancement. 1 **Initialization** 2 Generate $V^{\left[1\right]},\ldots ,V^{\left[M\right]}$ at legitimate BS and share transmit BF information with legitimate MSs  3 Broadcast a pilot signal at legitimate BS 4 Obtain $h_{\mathrm{MS},i}$, $h_{E,j}$ at legitimate MSs and eavesdroppers    
 5 **Generate of receive BF vectors**
 6 Generate $u_{\mathrm{MS},i}^{\left[m,b\right]}$ for all *m* and *b* at legitimate MSs  7 Generate $u_{E,j}^{\left[m,b\right]}$ for all *m* and *b* at eavesdroppers    
 8 **Feedback of SINR values**
 9 Feedback the SINRs $\Gamma_{\mathrm{MS},i}^{\left[m,b\right]}$ and corresponding *b* and *m* at legitimate MSs 10 Calculate the SINRs $\Gamma_{E,j}^{\left[m,b\right]}$ and corresponding *b* and *m* of eavesdroppers at legitimate BS    
11 **User scheduling** 12 Calculate $R_{\mathrm{MS}}^{\left[m\right]}$ and $R_{E}^{\left[m\right]}$ for all *m* at legitimate BS    
13 **Data transmission** 14 Transmit data signals x via $V^{\left[\hat{m}\right]}$ to maximize the secrecy sum-rate at legitimate BS 15 Achieve the secrecy sum-rate $R_{S}^{\left[\hat{m}\right]}$ for downlink data transmission at legitimate BS 


### 3.1. Initialization

*M* candidates of transmit BF matrix are generated by the legitimate BS in a *pseudo-random manner*. These candidates are shared with legitimate MSs. After that, to announce a wireless channel vector from the legitimate BS to legitimate MS, the legitimate BS broadcasts a pilot signal.

### 3.2. Generate of Receive Beamforming Vectors

Legitimate MSs who received a pilot signal generate MB receive BF vectors based on effective channel vector $h_{\mathrm{MS},i}$. Based on MMSE, the receive BF vector $u_{\mathrm{MS},i}^{\left[m,b\right]}$ corresponding to the *b*th transmit BF vector in the *m*th transmit BF matrix for all *m* and *b* is given by

(7) $$\begin{array}{c} u_{\mathrm{MS},i}^{\left[m,b\right]}=\frac{{\left(N_{0}I_{N_{R}}+R_{\mathrm{MS},i}^{\left[m,b\right]}\right)}^{-1}h_{\mathrm{MS},i}^{\left[m,b\right]}}{∥{\left(N_{0}I_{N_{R}}+R_{\mathrm{MS},i}^{\left[m,b\right]}\right)}^{-1}h_{\mathrm{MS},i}^{\left[m,b\right]}∥},\;\;\;\forall m,\;\forall b, \end{array}$$

where the interference covariance matrices $R_{\mathrm{MS},i}\in C^{N_{R}\times N_{R}}$ for all *m* and *b* are given by

(8) $$\begin{array}{c} R_{\mathrm{MS},i}^{\left[m,b\right]}=E\left[y_{\mathrm{MS},i}{\left(y_{\mathrm{MS},i}\right)}^{H}\right]-h_{\mathrm{MS},i}^{\left[m,b\right]}{\left(h_{\mathrm{MS},i}^{\left[m,b\right]}\right)}^{H}-N_{0}I_{N_{R}},\;\;\;\forall m,\;\forall b. \end{array}$$

Similar to the above procedure, by considering worst-case at legitimate devices, eavesdroppers also generate MB received BF vectors based on MMSE, which are given by

(9) $$\begin{array}{c} u_{E,j}^{\left[m,b\right]}=\frac{{\left(N_{0}I_{N_{R}}+R_{E,j}^{\left[m,b\right]}\right)}^{-1}h_{E,j}^{\left[m,b\right]}}{∥{\left(N_{0}I_{N_{R}}+R_{E,j}^{\left[m,b\right]}\right)}^{-1}h_{E,j}^{\left[m,b\right]}∥},\;\;\;\forall m,\;\forall b, \end{array}$$

where the interference covariance matrices $R_{E,j}\in C^{N_{R}\times N_{R}}$ for all *m* and *b* are given by

(10) $$\begin{array}{c} R_{E,j}^{\left[m,b\right]}=E\left[y_{E,j}{\left(y_{E,j}\right)}^{H}\right]-h_{E,j}^{\left[m,b\right]}{\left(h_{E,j}^{\left[m,b\right]}\right)}^{H}-N_{0}I_{N_{R}},\;\;\;\forall m,\;\forall b. \end{array}$$

### 3.3. Feedback of SINR Values

The SINR values at the *i*th legitimate MS for all *m* and *b* can be calculated by

(11) $$\begin{array}{c} \Gamma_{\mathrm{MS},i}^{\left[m,b\right]}=\frac{{\left|{\left(u_{\mathrm{MS},i}^{\left[m,b\right]}\right)}^{H}h_{\mathrm{MS},i}^{\left[m,b\right]}\right|}^{2}}{{\left(u_{\mathrm{MS},i}^{\left[m,b\right]}\right)}^{H}\left(N_{0}I_{N_{R}}+R_{\mathrm{MS},i}^{\left[m,b\right]}\right)u_{\mathrm{MS},i}^{\left[m,b\right]}},\;\;\;\forall m,\;\forall b. \end{array}$$

Similarly, the SINR values at the *j*th eavesdropper for all *m* and *b* can be calculated at the legitimate BS.

(12) $$\begin{array}{c} \Gamma_{E,j}^{\left[m,b\right]}=\frac{{\left|{\left(u_{E,j}^{\left[m,b\right]}\right)}^{H}h_{E,j}^{\left[m,b\right]}\right|}^{2}}{{\left(u_{E,j}^{\left[m,b\right]}\right)}^{H}\left(N_{0}I_{N_{R}}+R_{E,j}^{\left[m,b\right]}\right)u_{E,j}^{\left[m,b\right]}},\;\;\;\forall m,\;\forall b. \end{array}$$

We consider three types of feedback strategies: the conventional *full feedback* (F-FB), the conventional *opportunistic feedback* (O-FB) [26,27], and the proposed *codebook-based opportunistic feedback* (CO-FB) strategy. For the detailed explanations, we assume that the number of required bits to deliver the quantized SINR value is *Q* bits.

- In the conventional F-FB strategy [26,27], each legitimate MS provides a feedback of the maximal SINR value for all *m*. Hence, *M* SINR values are received back at the legitimate BS from each legitimate MS. Then, the number of required feedback bits per legitimate MS is represented as
  (13) $$\begin{array}{c} N_{F-\mathrm{FB}}=M\left(\lceil \log_{2}B\rceil +Q\right). \end{array}$$
  However, many feedback bits are required. Thus, we consider opportunistic feedback strategies for the reduction of feedback bits.
- In the conventional opportunistic feedback (O-FB) strategy [26,27], each legitimate MS selects $n\left(\leq M\right)$ maximal SINR values among *M* transmit BF vectors, where *n* is a *predetermined* value based on policy before data transmission. *n* SINR values and beam indices are received back from each legitimate MS. Then, the number of required feedback bits per legitimate MS is represented as
  (14) $$\begin{array}{c} N_{O-\mathrm{FB}}=n\left(\left\lceil \log_{2}MB\right\rceil +Q\right). \end{array}$$
- To further reduce the required feedback bits per legitimate MS, the proposed codebook-based opportunistic feedback (CO-FB) strategy can be applied instead of the conventional O-FB with one-time codebook sharing before data transmission. Each legitimate MS selects the maximal SINR in *n* transmit BF matrices out of *M* transmit BF matrix candidates. *n* SINR values and *n* codebook indices are received back from each legitimate MS. Then, the number of required feedback bits per legitimate MS is represented as
  (15) $$\begin{array}{c} N_{\mathrm{CO}-\mathrm{FB}}=\left\lceil \log_{2}\left(\begin{array}{c} M\\ n \end{array}\right)\right\rceil +n\left(\lceil \log_{2}B\rceil +Q\right). \end{array}$$

To compare the feedback amount for each feedback strategy, we consider the number of required feedback bits per legitimate MS when the number of candidates of transmit BF matrix *M* is equal to 16, the number of transmit BF vectors in each transmit BF matrix *B* is equal to 3, and the number of required bits for the quantized SINR *Q* is equal to 6 bits. In the conventional F-FB strategy, the number of required feedback bits per legitimate MS can be calculated by $N_{F-\mathrm{FB}}=16\left(\lceil \log_{2}3\rceil +6\right)=128$ bits. On the other hand, the number of required feedback bits per legitimate MS in the conventional O-FB strategy can be calculated as $N_{O-\mathrm{FB}}=4\left(\left\lceil \log_{2}16\times 3\right\rceil +6\right)=48$ bits when the predetermined valued is equal to n=4. Furthermore, in the proposed CO-FB strategy, the number of required feedback bits per legitimate MS can be calculated as $N_{\mathrm{CO}-\mathrm{FB}}=\left\lceil \log_{2}\left(\begin{array}{c} 16\\ 4 \end{array}\right)\right\rceil +4\left(\lceil \log_{2}3\rceil +6\right)=43$ bits.

### 3.4. User Scheduling

Since eavesdroppers’ CSI is available at the legitimate BS, the legitimate BS selects the optimal transmit BF matrix based on legitimate MSs’ feedback information and eavesdroppers’ CSI. In the first step, the legitimate BS selects a legitimate MSs with the maximal SINR value for all *b*. Then, the achievable sum-rate for all *m* is given by

(16) $$\begin{array}{c} R_{\mathrm{MS}}^{\left[m\right]}=\sum_{b=1}^{B}\left[\log_{2}\left(1+\max_{1\leq i\leq N_{\mathrm{MS}}}\Gamma_{\mathrm{MS},i}^{\left[m,b\right]}\right)\right],\forall m. \end{array}$$

Similarly, the eavesdropping rate due to eavesdropper for all *m* is given by

(17) $$\begin{array}{c} R_{E}^{\left[m\right]}=\sum_{b=1}^{B}\left[\log_{2}\left(1+\max_{1\leq j\leq N_{E}}\Gamma_{E,j}^{\left[m,b\right]}\right)\right],\forall m. \end{array}$$

### 3.5. Data Transmission

In the last step, the legitimate BS transmits a data signal vector x with the $\hat{m}$th optimal transmit BF matrix. Thus, the achievable secrecy sum-rate is obtained as

(18) $$\begin{array}{c} R_{S}^{\left[\hat{m}\right]}={\left(R_{\mathrm{MS}}^{\left[\hat{m}\right]}-R_{E}^{\left[\hat{m}\right]}\right)}^{+}. \end{array}$$

The achievable secrecy sum-rate can be obtained from both the achievable sum-rate in Equation (16) and the data-loss in Equation (17).

## 4. Simulation Results

We evaluated the conventional RBF and the proposed PRBF in MIMO downlink cellular network consisting of legitimate MSs and eavesdroppers according to various system parameters such as the number of transmit BF matrix candidates, the number of legitimate MSs, and the predetermined value in the conventional O-FB and the proposed CO-FB. The system parameter definitions are shown in Table 1. We also analyzed the number of required feedback bits at each legitimate MSs with all of considered feedback strategies.

**Figure 2:**
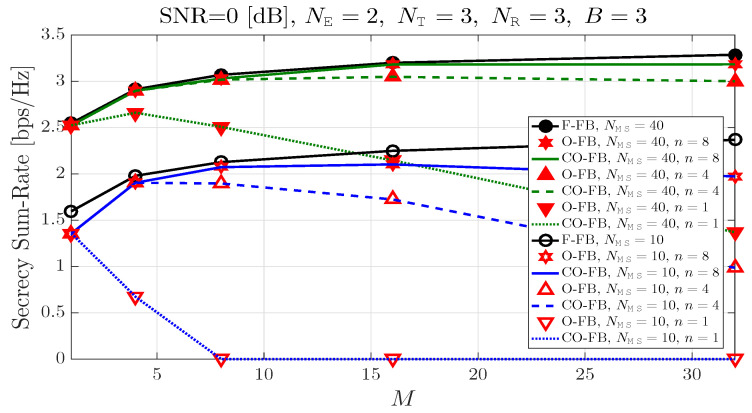
Secrecy sum-rate according to the number of BF candidates.

Figure 2 shows that the secrecy sum-rate according to the number of transmit BF matrix candidates *M* when the number of eavesdroppers $N_{E}$ is equal to 2, and the number of antennas at legitimate BS $N_{T}$, the number of antennas of both the legitimate MSs and eavesdroppers $N_{R}$ and the number of transmit BF vectors in each transmit BF matrix candidate *B* are all equal to 3. In addition, the received SNR at each communication device (legitimate MS or eavesdropper) is equal to 0dB. When the number of transmit BF matrix candidates is equal to 1, the curves show the secrecy sum-rate of the conventional PRBF. When the number of transmit BF matrix candidates is 2 or more, the curves show the secrecy sum-rate of the proposed PRBF. In addition, we consider the number of legitimate MSs $N_{\mathrm{MS}}=10$ and 40 cases in all of considered feedback strategies. The conventional O-FB and the proposed CO-FB show the same performance in terms of secrecy sum-rate with the same system parameters. Hence, we only consider the secrecy sum-rate of the proposed CO-FB in the following figures. The secrecy sum-rate increases as the number of transmit BF matrix candidates *M* increases in the conventional F-FB. In both the conventional O-FB and the proposed CO-FB, as the number of transmit BF matrix candidates *M* increases, the secrecy sum-rate does not always monotonically increase since the number of legitimate MSs $N_{\mathrm{MS}}$ and the predetermined value *n* are not large enough. However, when the predetermined value *n* or the number of legitimate MSs $N_{\mathrm{MS}}$ are large enough, the proposed CO-FB and the conventional F-FB show almost the same performance in terms of secrecy sum-rate.

**Figure 3:**
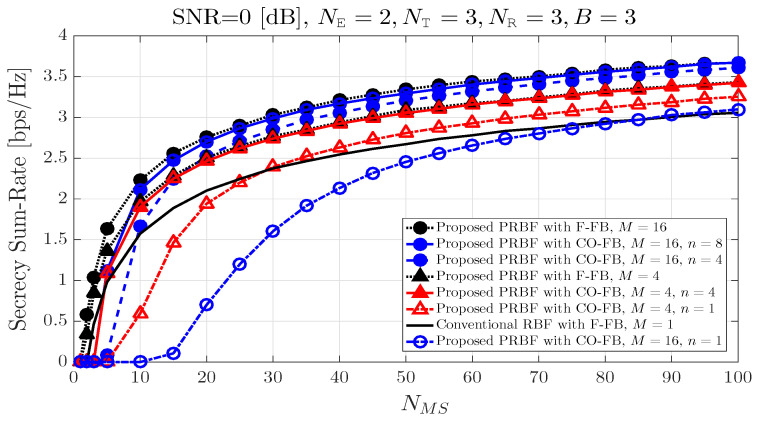
Secrecy sum-rate according to the number of legitimate MSs.

Figure 3 shows that the secrecy sum-rate according to the number of legitimate MSs $N_{\mathrm{MS}}$ when the number of eavesdroppers $N_{E}$ is equal to 2, and the number of antennas at legitimate BS $N_{T}$, the number of antennas at both of legitimate MSs and eavesdroppers $N_{R}$, the number of transmit BF vectors in each transmit BF matrix candidate *B* are all equal to 3. In addition, the received SNR at each communication device is equal to 0dB. In general, as the number of legitimate MSs $N_{\mathrm{MS}}$ increases, the secrecy sum-rate increases in the all of feedback strategies. The proposed CO-FB does not reach the performance of the conventional F-FB in terms of secrecy sum-rate when the predetermined value *n* or the number of legitimate MSs $N_{\mathrm{MS}}$ are not large enough. When the predetermined value *n* or the number of legitimate MSs $N_{\mathrm{MS}}$ are large enough, the proposed CO-FB and the conventional F-FB show almost the same performance in terms of secrecy sum-rate.

**Figure 4:**
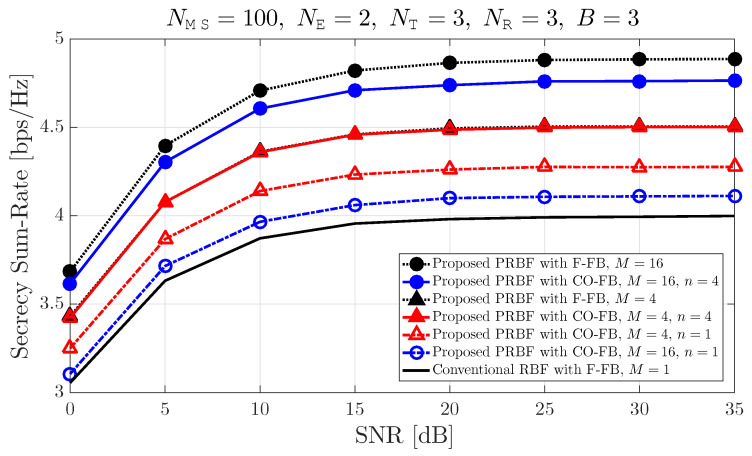
Secrecy sum-rate according to the received SNR.

Figure 4 shows that the secrecy sum-rate according to the received SNR at each communication device when the number of legitimate MSs $N_{\mathrm{MS}}$ is equal to 100, snf the number of eavesdroppers $N_{E}$ is equal to 2, the number of antennas of both legitimate MSs and eavesdroppers $N_{R}$, and the number of transmit BF vectors in each transmit BF matrix candidate *B* are all equal to 3. The secrecy sum-rate increases as the received SNR and the number of transmit BF candidates *M* increases. In particular, when the number of transmit BF candidates *M* is equal to 4, the proposed PRBF with the conventional F-FB outperforms the proposed PRBF with the proposed CO-FB and the predetermined value n=1 in terms of secrecy sum-rate. However, the proposed PRBF with the conventional F-FB and the proposed PRBF with the proposed CO-FB and the predetermined value n=4 are almost the same in terms of secrecy sum-rate. When the predetermined value *n* is large enough, the proposed CO-FB and the conventional F-FB show almost the same performance in terms of secrecy sum-rate. However, when the predetermined value *n* increases, the number of required feedback bits per legitimate MS also increases.

**Figure 5:**
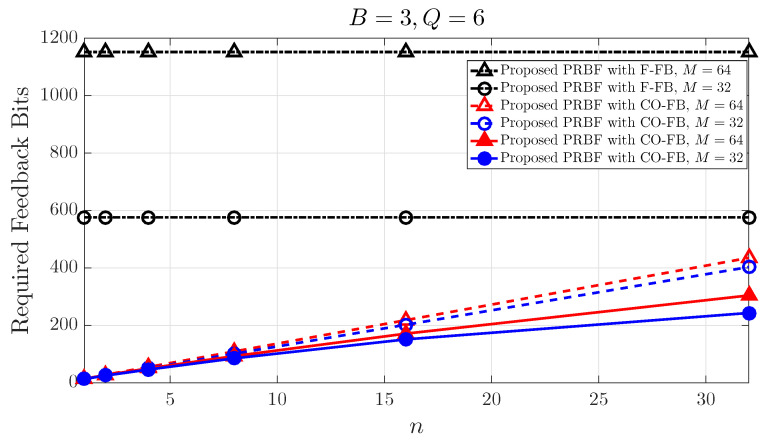
Required feedback bits per legitimate MS according to the predetermined value.

Figure 5 shows that the number of required feedback bits per legitimate MS according to the number of transmit BF matrix candidates *M* and the predetermined value *n* when the number of transmit BF vectors in each transmit BF matrix candidate *B* is equal to 3, and the number of required bits for SINR quantization *Q* is equal to 6 bits. As explained in Section 3.2, each legitimate MS provides back totally *M* SINR values and beam indices in the conventional F-FB. In the conventional F-FB, the number of required feedback bits do not depend on the predetermined value *n*. Thus, the number of required feedback bits is fixed regardless of the predetermined value *n*. When the number of transmit BF matrix candidates *M* is equal to 16 and the number of transmit BF vectors in each transmit BF matrix candidate *B* is equal to 3 in the conventional F-FB strategy, each legitimate MS provides back totally 16 SINR values for 16 BF matrix candidates regardless of *n*. On the other hand, each legitimate MS provides back totally *n* SINR values and beam indices in both of the conventional O-FB and the proposed CO-FB. The predetermined value *n* indicates the number of feedback SINR values, and the number of required feedback bits depends on the predetermined value *n* in both of the conventional O-FB and the proposed CO-FB. When the predetermined value *n* and the number of transmit BF vectors in each transmit BF matrix candidate *M* are equal to 4 and 16, respectively, each legitimate MS provides back only four maximal SINR values for 16 BF matrix candidates in the conventional O-FB. Furthermore, in the proposed CO-FB, the number of required feedback bits per legitimate MS can be reduced compared to the conventional O-FB. The conventional F-FB shows the best performance than the proposed CO-FB in terms of secrecy sum-rate. However, it is difficult to apply the conventional F-FB in practice due to the large number of required feedback bits. Hence, the proposed CO-FB maintains a similar performance as the conventional F-FB in terms of secrecy sum-rate when the number of legitimate MSs, the number of transmit BF matrix candidates *M* and the predetermined value *n* are large enough. As a result, the feedback overhead can be significantly reduced.

In summary, the conventional PRBF uses only one pseudo-random BF matrix (M=1) for downlink data transmission. Accordingly, each legitimate MS feedbacks SINR values with transmit BF vector index to the legitimate BS according to the number of transmit BF vectors *B*. However, the proposed PRBF uses one more pseudo-random BF matrix candidates (M≥2). Thus, each legitimate MS feedbacks SINR values with transmit BF vector and matrix index to the legitimate BS according to the number of transmit BF matrix candidates *M* and the number of transmit BF vectors *B*. The proposed PRBF technique has the effect of increasing the number of legitimate MSs who feedback CSI by the number of transmit BF matrix candidates *M*. In other words, the proposed PRBF technique exploits *multi-user diversity gain*. As the number of transmit BF matrix candidates *M* increases, the sum-rate increases, however, the feedback overhead also significantly increases in the proposed PRBF with the conventional F-FB. Thus, the proposed PRBF technique should be used with the conventional O-FB or the proposed CO-FB, which can significantly reduce the feedback overhead. However, in the proposed PRBF with the conventional O-FB or the proposed CO-FB, if the number of transmit BF matrix candidates *M* is large and the number of legitimate MSs $N_{\mathrm{MS}}$ is small, the sum-rate is reduced because some transmit BF vectors may not be selected and used during user scheduling. To solve this problem, the predetermined value *n* should be designed considering the number legitimate MSs $N_{\mathrm{MS}}$ in a downlink cell to obtain a sufficient sum-rate improvement.

## 5. Conclusions

In this paper, we propose a PRBF technique for MIMO downlink cellular networks. Legitimate MSs can receive the data signal from the legitimate BS via MMSE-based receive BF vector. By considering worst-case at legitimate devices, we assume that potential eavesdroppers can also receive the data signal via MMSE-based receive BF vector. Based on the feedback information from legitimate MSs and potential eavesdroppers’ CSI, the legitimate BS selects the optimal transmit BF matrix among multiple BF candidates in order to maximize the secrecy sum-rate performance. Extensive computer simulations show that the proposed PRBF outperforms the conventional RBF in terms of secrecy sum-rate. In addition, the proposed CO-FB and the conventional O-FB have the same performance in terms of secrecy sum-rate; however, the proposed CO-FB outperforms the conventional O-FB in terms of required feedback bits. Furthermore, when the number of legitimate MSs and the predetermined value are large enough, the proposed PRBF with the proposed CO-FB outperforms the conventional RBF with the conventional O-FB in terms of sum-rate and required feedback bits for user scheduling at the legitimate BS.

## Figures and Tables

**Table 1:** System parameters.

Definition	Parameter
Number of antennas at the legitimate BS	$N_{T}$
Number of antennas at the legitimate MSs and eavesdroppers	$N_{R}$
Number of legitimate MSs	$N_{\mathrm{MS}}$
Number of eavesdroppers	$N_{E}$
Number of transmit BF matrix candidates	*M*
Number of transmit BF vectors in each BF matrix candidate	$B(=N_{T})$
Number of bits for SINR quantization	*Q*
Predetermined value in O-FB and CO-FB	*n*
